# Intraabdominelle Gefäßverletzungen nach stumpfem Bauchtrauma

**DOI:** 10.1007/s00104-023-01931-9

**Published:** 2023-07-20

**Authors:** D. Böckler, J. Hatzl, M. S. Bischoff, De Hua Chang, K. Meisenbacher, A. Peters

**Affiliations:** 1 Klinik für Gefäßchirurgie und Endovaskuläre Chirurgie, Universitätsklinikum Heidelberg, Im Neuenheimer Feld 420, 69120 Heidelberg, Deutschland; 2 Klinik für Radiodiagnostik und Interventionelle Radiologie, Universitätsklinikum Heidelberg, Heidelberg, Deutschland

**Keywords:** Focused assessment with sonography for trauma, Offene Techniken, Endovaskuläre Techniken, Verletzungsmechanismus, Antikoagulation, Focused assessment with sonography for trauma, Open techniques, Endovascular techniques, Injury mechanism, Anticoagulation

## Abstract

Gefäßverletzungen und konsekutive Blutungen sind ernsthafte potenzielle Komplikationen bei der Behandlung von Patienten mit stumpfem Bauchtrauma. Die Behandlung hängt vom Ausmaß und der Lokalisierung ab und kann von der Überwachung über die endovaskuläre Behandlung bis hin zur offenen Operation reichen. Der Schlüssel zum Erfolg sind das FAST(„focused assessment with sonography for trauma“)-Management und eine frühzeitige Entscheidungsfindung. Das abdominale Gefäßtrauma ist nach wie vor ein schwieriges Problem und die offenen und endovaskulären Techniken entwickeln sich weiter, um diesen komplexen Krankheitsprozess zu behandeln.

## Abstract (en)

Vascular injuries and hemorrhaging are serious potential complications in the management of patients with blunt abdominal trauma. The treatment depends on the extent and localization and can range from surveillance to endovascular treatment up to open surgery. The keys to success include the focused assessment with sonography for trauma (FAST) management and timely decision making. Abdominal vascular trauma continues to be a difficult problem and open and endovascular techniques continue to evolve in order to address this complex disease process.

## Hintergrund

Intraabdominelle oder retroperitoneale Blutungen durch Verletzungen von Gefäßen stellen im Rahmen eines stumpfen Bauchtraumas (sBT) eine potenziell schwerwiegende Komplikation dar. Laut einer aktuellen systematischen Literaturrecherche beträgt die allgemeine Sterblichkeit nach sBT 28 %, die Letalität nach offener Operation 13,5 % [1]. 12,2 % der Patienten erleiden eine Aortenruptur, die zweite Gruppe stirbt am schockbedingten Multiorganversagen [2].

Das Management des stumpfen Abdominaltraumas erfordert neben chirurgischer bzw. gefäßchirurgischer und interventioneller Expertise auch Kenntnisse über perioperative Behandlungskonzepte z. B. hinsichtlich des Gerinnungsmanagements [3]. Heutzutage stehen in der Gefäßchirurgie multimodale Behandlungstechniken zur Verfügung: Neben den etablierten klassischen chirurgischen Operationstechniken beinhalten diese mittlerweile minimal-invasive endovaskuläre Techniken wie z. B. Coil-Embolisation oder die perkutane Implantation gecoverter Stents (Stentgrafts). Die Kombination dieser Techniken wird als Hybridoperation bezeichnet und kann entweder in Kooperation mit interventionellen Radiologen oder eigenständig durchgeführt werden. Voraussetzungen hierfür sind eine entsprechende Ausstattung des Operationssaales mit Durchleuchtungs- und Angiographieeinheit, im Idealfall ein Hybridoperationsraum, des Weiteren aber auch Kenntnisse in Materialkunde und Indikationsstellung zur Verfahrenswahl und die 24 h/7 Tage-Verfügbarkeit der notwendigen operativen Expertise. Bisweilen sind zwei- bzw. mehrzeitige Operationen nach abdominellen Tamponaden notwendig und sinnvoll. Vor diesem Hintergrund entscheidet oft die chirurgische Strategie und das übergeordnete Behandlungskonzept über das operative Ergebnis und das Überleben des Patienten [4].

Stumpfe und v. a. penetrierende Abdominaltraumata sind häufig mit Gefäßverletzungen assoziiert. Gefäßverletzungen sind hierbei die häufigste Todesursache nach penetrierenden Verletzungen des Abdomens und stellen für das Notfallteam und den/die ChirurgIn eine hohe operative Herausforderung dar. Für das Überleben des Patienten sind eine frühzeitige Erkennung des Verletzungsmusters, eine schnelle Verbringung in ein Haus der Maximalversorgung und eine interdisziplinäre Einschätzung und weitere Versorgung entscheidend. Nach stumpfem Abdominaltrauma stellen sich die klinische Situation des Patienten und das Verletzungsmuster anders als bei penetrierenden Verletzungen dar.

Der folgende Übersichtsartikel fokussiert aus gefäßchirurgischer Sicht auf die Verletzungsmechanismen, die Diagnostik, Therapie und das perioperative Management begleitender Gefäßverletzungen im Rahmen eines stumpfen Bauchtraumas.

## Typen des Verletzungsmechanismus

Für begleitende Gefäßverletzungen nach stumpfen Bauchtrauma sind 3 verschiedene Verletzungsmechanismen bekannt [5]:

1. Das schnelle Dezelerationstrauma z. B. nach Verkehrsunfällen mit Hochgeschwindigkeitsauffahrunfällen im Straßenverkehr (KFZ, Motorrad) – vergleichbar dem traumatischen Aortenabriss der thorakalen Aorta [5] – oder dem Sturz aus großer Höhe (Unfall, suizidale Absicht). Hierbei kommt es potenziell zur Verletzung der Viszeralarterien, selten der abdominellen Aorta mit Intimaeinriss, mit Dissektion und Avulsion der Gefäßwand. Intramurale Hämorrhagien oder transmurale Blutungen sowie konsekutive arterielle Thrombosen sind die potenziellen Folgen. Klinisch folgen der arteriellen Verletzung entsprechend Schock oder Organ-Bein-Ischämien.
2. Direkte anteroposteriore Quetschung von KFZ-Insassen durch den querverlaufenden Anteil des Sicherheitsgurtes oder direkter Schlag auf die vordere Bauchwand.
3. Direkte Gefäßlazeration größerer abdomineller Gefäße durch Knochenfragmente bei ausgedehnten Beckenringfrakturen.

Dabei können arterielle und venöse Gefäßverletzungen in gleicher Häufigkeit auftreten.

Der Vollständigkeit halber sei erwähnt, dass das penetrierende Abdominaltrauma für die meisten abdominellen Gefäßverletzungen verantwortlich ist und ca. 90 % aller Behandlungsfälle in Traumazentren, Unfallkliniken und Schockraumbehandlungen darstellt.

Die Vena cava inferior ist am häufigsten betroffen 

In einer Single-Center-Studie aus den USA [6] von 302 Patienten mit abdominellen traumatischen Gefäßverletzungen betrug die Inzidenz für venöse Verletzungen 51 % und für arterielle Verletzung 41 %. Dabei war in 1 von 4 Fällen das am häufigsten verletzte Gefäß die Vena cava inferior, gefolgt von der abdominellen Aorta, den Beckenarterie und Beckenvenen und den Mesenterialgefäßen (Tab. 1).

**Table 1:** 

Reihenfolge	Betroffenes Gefäß	Häufigkeit (%)
1	Vena cava inferior	25
2	Abdominelle Aorta	21
3	Beckenarterien	20
4	Beckenvenen	17
5	Vena mesenterica superior	11
6	Arteria mesenterica superior	10

Betont werden muss, dass mit durchschnittlich 1,6 betroffenen Gefäßen meist ein multilokuläres Verletzungsmuster vorliegt, sodass man bei der computertomographischen (CT-)Angiographie und/oder sofortigen abdominellen Exploration explizit nach nicht erkannten weiteren Gefäßverletzungen suchen muss.

## Verletzungsmechanismus: anatomisch-chirurgische Aspekte

**Figure 1:**
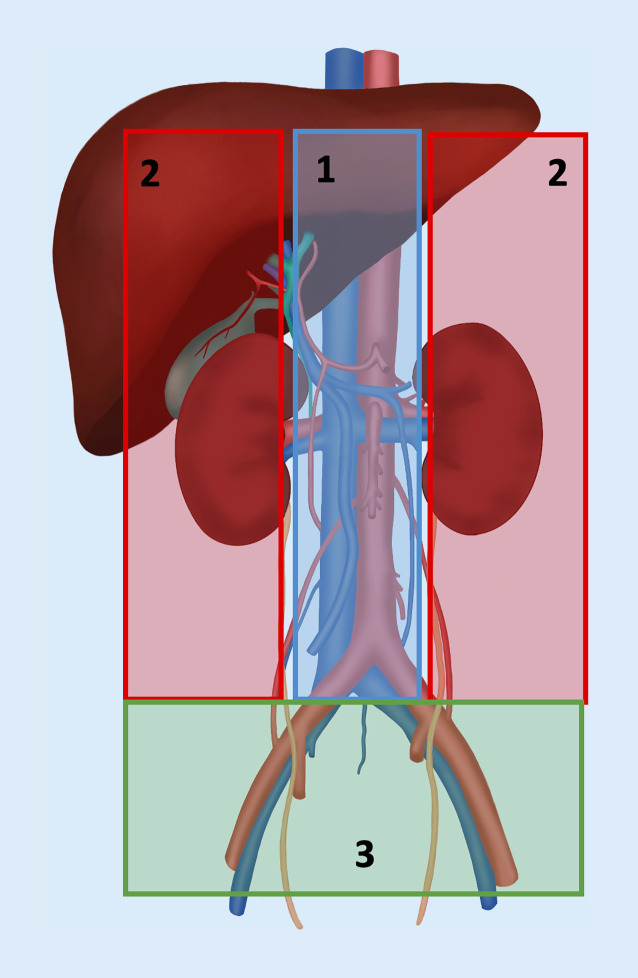

Bei den differenzialdiagnostischen Überlegungen hinsichtlich evtl. begleitender Gefäßverletzungen nach stumpfem Bauchtrauma hilft die topgrafische Lokalisation der Verletzung bzw. des Traumas. Man unterscheidet dabei drei topographische Zonen im Abdomen inkl. Retroperitonealraum (Abb. 1). Entsprechend der dort lokalisierten vaskulären Strukturen können unterschiedliche Gefäßverletzungsmuster vorliegen und entscheiden damit über die chirurgische Strategie (offen, endovaskulär) und über den Gefäßzugang (offen, perkutan) und ggf. auch über die durchführende Disziplin(en).

## Mechanismen abdomineller Gefäßverletzungen

Stumpfe Aortenverletzungen sind meist Folge einer Dezeleration oder Kompression.

Die Gewalt bei Dezeleration wirkt von kaudal-ventral nach dorsokranial. Dies löst einen sog. Schaufelmechanismus aus, der über eine axiale Zerrung der Aorta oder Abkantung bei Wirbelfrakturen zur Aortenverletzung führt. Die Kompression entsteht meist bei Überolltraumen im Straßenverkehr.

Aortale Verletzungen werden anhand ihres Verletzungsmusters im CT klassifiziert:

- Grad 1: Intimalazeration ohne/mit Thrombus < 10 mm,
- Grad 2: große Intimalefze > 10 mm,
- Grad 3: Pseudoaneurysma,
- Grad 4: Ruptur.

Verletzungen der Art. mesenterica inferior sind eine absolute Rarität. Verletzungen der Art. mesenterica superior im Rahmen eines stumpfen Bauchtraumas sind bei Dezelarations- und/oder Kompressionstraumen häufiger und bedürfen einer meist offen-chirurgischen Therapie [9].

## Klinische Präsentation und diagnostische Evaluation

Viele Patienten mit großen, multiplen und schwerwiegenden Gefäßverletzungen nach stumpfem Bauchtrauma erreichen das weiterversorgende Krankenhaus nicht und sterben mit oder ohne notfallmedizinische Erstmaßnahmen am Unfallort. 14 % der Patienten versterben auf dem Transportweg zum Krankenhaus.

Die klinischen Symptome hängen vom verletzten Gefäß (Vene, Arterie), dem Verletzungsausmaß (Polytrauma) und der verstrichenen Zeit zwischen Unfall und Versorgung ab. Schockparameter, abdominelle Distension, insbesondere wenn nicht responsiv auf i.v. Volumengabe – sind richtungsweisend. Normotension und vermeintliche Kreislaufstabilität können dabei irreführend sein oder falsche Sicherheit vortäuschen. Retroperitoneale Hämatome oder gedeckte Gefäßrupturen sowie Tamponaden venöser Blutungen bleiben meisten in der Prähospitalphase unerkannt.

Normotension und vermeintliche Kreislaufstabilität können falsche Sicherheit vortäuschen

Asymmetrische Pulse sind mögliche Erstsymptome bei Beckenarterienverschlüssen durch Dissektion mit oder ohne thrombotischen arteriellen Verschluss. Gefäßverletzungen im Rahmen stumpfer abdomineller Traumata werden häufig bei initialer Untersuchung und Erstversorgung am Unfallort oder sogar im Krankenhaus übersehen, falls keine Hämorrhagie oder Ischämie vorliegt. „Daran denken“ ist entscheidend.

Der Begriff „focused assessment with sonography for trauma“ (FAST) beschreibt einen standardisierten sonographischen Untersuchungsgang des Bauches und Beckens und in der Erweiterung auch der Brusthöhle beim schwerverletzten Patienten im Schockraum [10]. Die FAST-Untersuchung kommt regelmäßig bei der Initialphase der Untersuchung von Polytraumapatienten im Schockraum zur Anwendung. Ziel der FAST-Untersuchung des Schockraumpatienten ist das Erkennen freier Flüssigkeit als Zeichen einer Verletzung von Organen und Blutgefäßen des Bauchraumes und damit gegebenenfalls die Entscheidung zu sofortigen lebensrettenden Operationen. Ihre Sensitivität und Spezifität betrug dabei in prospektiven Studien nahezu 100 %.

Die CT-Angiographie mit Kontrastmittelunterstützung (CT-A) stellt nach initialer abdomineller Sonographie zum Ausschluss freier Flüssigkeit die entscheidende diagnostische Maßnahme dar. Wichtig ist das CT-Protokoll mit Dünnschichtaufnahmen („slice thickness“ 0,75–1 mm), drei Ebenen (axial, sagittal, koronar) und die Durchführung einer frühen arteriellen und – wichtig – einer venösen späten Phase. Venöse retroperitoneale Blutungen werden sonst übersehen.

## Management traumatisch bedingter arterieller und venöser Blutungen

Primäres Ziel der Blutstillung und der Reparatur arterieller und venöser Blutung ist zunächst die Vermeidung des hämorrhagischen Schocks und dessen Folgen (Multiorganversagen, Gerinnungsstörung, Tod). Patienten, die bei penetrierenden Verletzungen mit hämodynamischer Instabilität, Peritonitis und Pneumoperitoneum bei stumpfem Bauchtrauma eine positive FAST-Untersuchung aufzeigen, müssen notfallmäßig offen exploriert werden.

Im Rahmen der offen-chirurgischen Blutungskontrolle sind Kenntnisse gefäßchirurgischer Gefäßklemmungen und entsprechende Nahttechniken hilfreich bzw. eine wichtige Voraussetzung für eine schnelle, suffiziente und andauernde Rekonstruktion von Gefäßverletzungen. Am Rande sei hier auf ein Aus- und Weiterbildungsdefizit in diesem Gebiet im Rahmen der aktuellen Weiterbildungsordnung mit „common trunk“ und früher Spezialisierung hingewiesen. Eine gefäßchirurgische Rotation ist aus Sicht der Autoren insbesondere für Chirurginnen/-chirurgen mit dem Schwerpunkt Tumorchirurgie und/oder Transplantationschirurgie sehr empfehlenswert.

Bei potenziell bakteriell kontaminiertem Situs sollte autologes Ersatzmaterial eingesetzt werden

Die Verletzung viszeraler Hauptstammarterien (Arteria mesenterica superior [AMS], Truncus coeliacus [TC]) ist im Rahmen stumpfer Bauchtraumata extrem selten. Bei unübersichtlichem Situs und lokal unkontrollierbarer Blutung kann nach Spaltung des Omentum minus und beider Zwerchfellschenkel eine subdiaphragmale bzw. supratrunkale Aortenklemmung notwendig und sinnvoll sein [9]. Als Gefäßrekonstruktionen kommen je nach Ausdehnung die Direktnaht, die Patchplastik oder das Interponat infrage. Bei potenziell bakteriell kontaminiertem Situs (z. B. begleitende Hohlorganperforation) sollte auf autologes Ersatzmaterial, z. B. mit Vena saphena magna oder Rinderperikard, zurückgegriffen werden. Alloplastisches Material (Polyester Dacron, Polytetrafluorethylen [PTFE]) führt in letzterem Fall zum Infekt und sollte primär, wenn möglich, vermieden werden. Sollte eine alloplastische Rekonstruktion z. B. aus Gründen eines Kalibermismatches des zu rekonstruierenden Gefäßes notwendig werden, ist auf eine sorgfältige Retroperitonealisierung zu achten, um sekundäre Protheseninfekte zu vermeiden. In scheinbar ausweglosen Situationen und lebensbedrohlichem Schockzustand kann die primäre Ligatur des TC oder der AMS gerechtfertigt sein. Eine simultane Unterbindung beider Gefäße verbietet sich [11]. Ob die gastroduodenale Arkade zwischen AMS und TC zur Perfusion von Leber und Magen bei Ligatur des TC ausreicht, ist schwer bzw. nicht zu prognostizieren. Eine intraoperative Duplexsonographie der Leberpforte kann hier weiterhelfen. Eine zeitnahe Second-look-Operation (< 48 h) ist notwendig, um potenzielle Organischämien rechtzeitig zu erkennen.

## Venöse Blutungen

Venöse Blutungen werden hinsichtlich des Blutverlustes und ihrer Hämodynamik häufig unterschätzt. Die Mortalität der V.-cava-inferior-Verletzung im Rahmen traumatischer Begleitverletzungen hat bei unverzüglicher Versorgung eine bessere Prognose als die traumatische Verletzung der unteren Hohlvene, die mit einer Sterblichkeit von 50 % assoziiert ist [12].

Die Dünnwandigkeit von Venen kann das Verletzungsausmaß schnell vergrößern

Primäres Ziel bei der Verletzung großer Venen ist die Blutungskontrolle und sekundär, wenn möglich, die Rekonstruktion. Ggf. ist die Ligatur notwendig und indiziert, die in der Regel durch venöse Kollateralkreisläufe gut kompensiert wird [11]. Primär ist die übersichtliche Präparation, Exploration und gute Übersicht eine wichtige Grundvoraussetzung. Technisch stehen die temporäre Tamponade, die Ligatur oder Rekonstruktion mittels Direktnaht, Patchplastik oder Interponat als alternative Verfahren zur Verfügung. Venöse Blutungen werden hinsichtlich des zu erwartenden Blutverlustes häufig unterschätzt, sodass eine frühzeitige Verwendung des Cell Saver® (Cell Saver® *Elite*®, Haemonetics®, München, Deutschland) empfohlen ist.

Venöse Blutungen sind aus gefäßchirurgischer Sicht anspruchsvoller und bei großlumigen Gefäßen aus Sicht der Autoren gefährlicher. Dies liegt an den zahlreichen zufließenden Ästen und dem komplexen Ein- und Ausstromnetzwerk, was die proximale und distale Gefäßkontrolle schwieriger macht. Die Dünnwandigkeit von Venen und die chirurgische Manipulation können das Verletzungsausmaß schnell vergrößern. Kleine venöse Blutungen können durch die Applikation hämostyptischer Agenzien oder „geduldiger“ Kompression mit Kompressen oder Bauchtüchern (ggf. temporär als Packing) erfolgreich kontrolliert werden. Ligaturen von Venen bleiben im Gegensatz zu Arterien aufgrund ihres Kollateralnetzwerkes oft folgenlos und können somit liberaler durchgeführt werden [13].

## Das REBOA-Manöver

**Figure 2:**
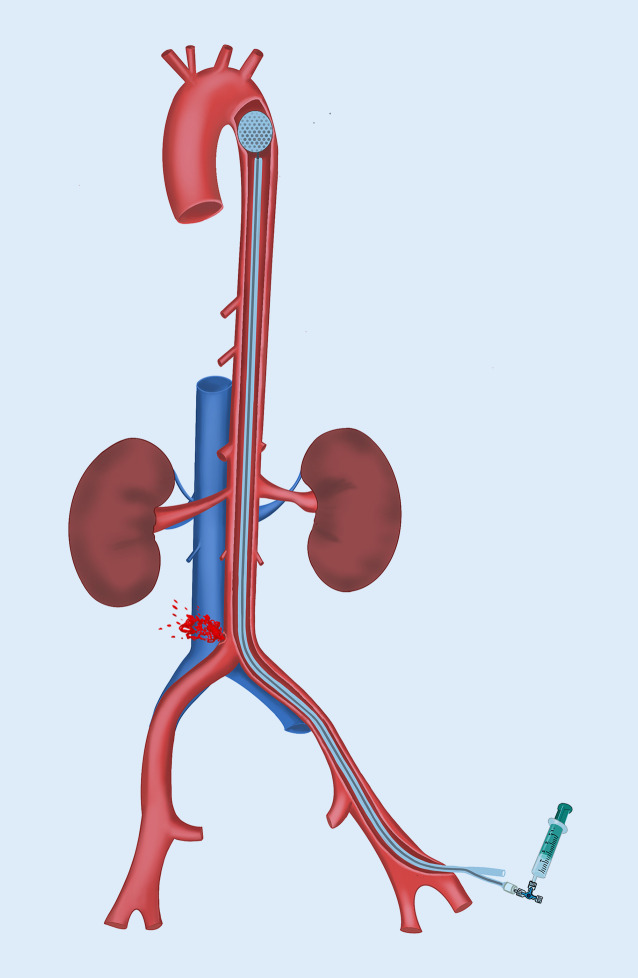

Das REBOA-Manöver („resuscitative endovascular balloon occlusion of the aorta“) wurde erstmalig im Jahre 2005 im Rahmen der Therapie des rupturierten abdominellen Aortenaneurysmas (AAA) beschrieben [14]. Dabei wurde ein Ballon perkutan transfemoral in die suprarenale Aorta proximal der Rupturstelle platziert und unter Sicht und Einsatz von Kontrastmittel inflatiert (Abb. 2). Um den Okklusionsballon allerdings in dieser Position zu halten, war die Einbringung einer großen Schleuse (> 14 Fr) zwingend notwendig. Ansonsten käme es zur Dislokation mit der aortalen Pulswelle, selbst bei schockbedingter Hypotonie. Für die Platzierung einer Schleuse ist wiederum ein steifer Führungsdraht notwendig, der im Idealfall unter Durchleuchtungskontrolle (Röntgenstrahlen) endovaskulär eingeführt wird. Eine detaillierte Beschreibung, wann und wie ein Okklusionsballon in der Aorta – zumindest aus gefäßchirurgischer Sicht – Sinn macht, wurde vom Erstbeschreiber der Technik, von Martina Malina aus Malmö publiziert [14]. Für weitere Informationen zur Durchführung wird auf die deutschsprachige Publikation von Wortmann et al. verwiesen [15].

Der Ballon sollte wegen des Risikos der via falsa nicht „blind“ vorgeschoben werden

Zahlreiche Fallbeispiele belegen den lebensrettenden Effekt von REBOA in selektionierten Fällen [16]. Vor dem Hintergrund der in diesen Veröffentlichungen beschriebenen technischen und prozeduralen Details ist das Prinzip und Konzept des REBOA-Manövers aus Sicht des (endovaskulär erfahrenen) Gefäßchirurgen vielversprechend und naheliegend, in der Umsetzung und Praxis aber nicht immer einfach und nicht ungefährlich. Folgende „Caveats“ sind aus Sicht des Autors zu berücksichtigen:

- Patienten im hämorrhagischen Schock, insbesondere Patienten jungen Alters, weisen spastische Femoral- und Beckenachsen auf, sodass die Gefäßpunktion in der Leistenregion unter Ultraschallkontrolle empfohlen wird.
- Die bloße Einführung des relativ steifen Ballonsystems kann durch physiologisch gekinkte/gewinkelte Beckengefäße erschwert bis unmöglich werden und ist mit einem Perforationsrisiko verbunden.
- Führungsdrähte ermöglichen den Zugang und Vorschub des Ballons, sollten aber ohne Sichtkontrolle wegen des Risikos der via falsa (z. B. in Nieren- oder Mesenterialarterien) nicht „blind“ vorgeschoben werden.
- Ohne Draht keine Schleuse und ohne Schleuse keine stabile Ballonpositionierung!

Somit ist einschränkend festzuhalten, dass der Einsatz im prähospitalen Notfall aktuell mit Einschränkungen möglich ist [17]. Zukünftige spezifische REBOA-Systeme werden diese technische Limitation ggf. überwinden. Eine aktuelle Publikation von Okada et al. beschreibt auch erste technische Platzierungserfolge ohne Röntgenstrahlen [18].

Nicht zuletzt ist die Lokalisation einer vermeintlichen aortalen Blutungsquelle nach stumpfem oder penetrierendem Bauchtrauma im Notfallsetting klinisch nicht immer eindeutig definierbar und die Füllmenge des Ballons bei unbekanntem Aortendurchmesser nicht messbar. Aus gefäßchirurgischer Sicht ist REBOA insbesondere im Krankenhaus (Schockraum, Operationssaal) ein sinnvolles minimal-invasives Konzept, um Patienten im Schock bei Gefäßverletzungen zu stabilisieren. Der interdisziplinäre Einsatz von REBOA gehört somit zum Armamentarium der Blutungskontrolle aortaler oder arteriell-iliakaler Gefäßverletzungen.

## Management antikoagulierter Patienten bei stumpfem Bauchtrauma

Neben invasiven Therapiemaßnahmen sind Kenntnisse der physiologischen Gerinnung der Antikoagulation und ihrer Antagonisierung notwendig [3]. Ein weit verbreitetes Antikoagulans ist Phenproucom (Marcumar). Marcumarisierte Patienten sollten in Anlehnung an den aktuellen INR (International Normalized Ratio) und dem Verdacht auf eine etwaige Blutung gemäß den ACCP(American College of Clinical Pharmacy)-Empfehlungen 2001 [19] vorsichtshalber wie folgt antagonisiert werden:

1. INR < 5: keine Blutung – Reduktion oder Einstellung der nächste Marcumar-Gabe,
2. INR 5 < 9: Einstellung der nächste Marcumar-Gabe, Vitamin K oral (2,5–5,0 mg),
3. INR > 9 ohne Blutung. Marcumar-Stopp, Vitamin K oral (2,5–5,0 mg) 4 FFP(„fresh frozen plasma“)-Konzentrate,
4. Blutung bei jeglicher INR: FFP, Vitamin K 10 mg langsam i.v., ggf. rekombinierter Faktor 7a.

Traumapatienten unter Marcumar, die binnen 24–48 h operiert werden müssen, werden wie folgt anatgonisiert:

1. INR < 2,0: Vitamin K 1,0 mg p.o.,
2. INR 2–5: Vitamin K 1–2,5 mg p.o.,
3. INR 5–9: Vitamin K 2,5–5,0 mg p.o., erneute Gabe von 1–2 mg nach 24 h, wenn INR weiterhin erhöht.

Bezüglich der exakten Dosierung sei auf die Begleitinformation der jeweiligen Medikamente verwiesen.

## Klinische Fallbeispiele von Gefäßverletzungen im Rahmen stumpfer abdomineller Gefäßverletzungen

### Kasuistik 1.

**Figure 3:**
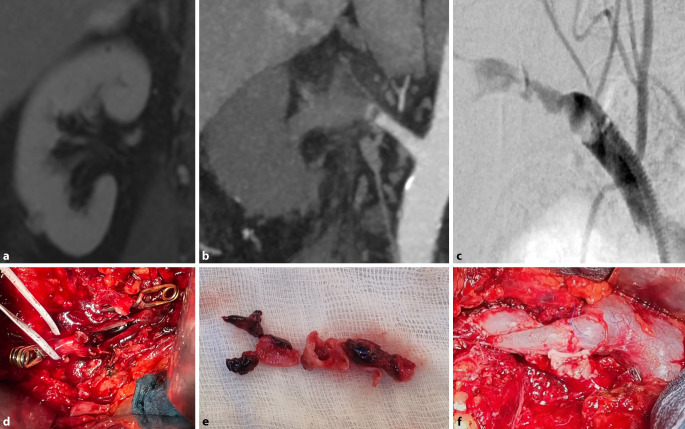

Ein vierzehnjähriges Mädchen erleidet ein stumpfes Bauchtrauma in Zone 2 rechts durch den Fahrradlenker nach Sturz. Die CT-Angiographie zeigt eine minderperfundierte rechte Niere (Abb. 3a) und einen Kontrastmittelabbruch und Verschluss der rechten Nierenarterie (Abb. 3b). Die diagnostische Angiographie in Interventionsbereitschaft bestätigt den thrombotischen Verschluss mit Verdacht auf eine Dissektion (Abb. 3c). Im Rahmen der offen-chirurgischen Konversionsoperation bestätigt sich die Dissektion (Abb. 3d). Nach Entfernung von Thrombus und Dissektat (Abb. 3e) erfolgt die Revaskularisation mittels boviner Patchplastik (Abb. 3f).

### Kasuistik 2.

**Figure 4:**
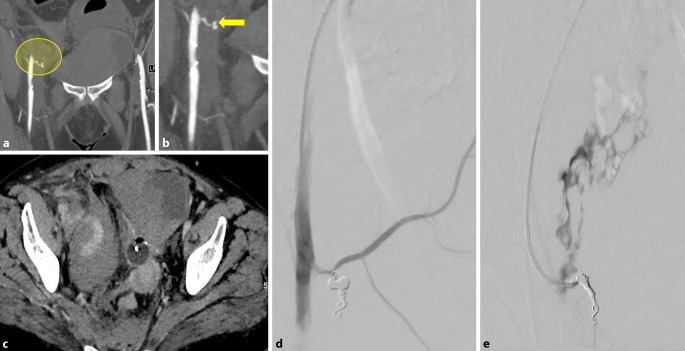

Die CT-Angiographie und digitale Substraktionsangiographie (DSA) zeigen eine arterielle Blutung im kleinen Beckenbereich aus der Arteria obturatoria. Sie wurde mittels Coil-Embolisation interventionell/endovaskulär erfolgreich gestillt (Abb. 4).

### Kasuistik 3.

Ein fünfzigjähriger Mann erleidet ein Dezelerationstrauma im Rahmen eines KFZ-Auffahrunfalles. Der Sicherheitsgurt verursachte ein stumpfes, querverlaufendes Unterbauchtrauma. Der Patient ist kreislaufstabil Die CT-Angiographie zeigt eine isolierte abdominelle Aortendissektion, die konservativ mit 3 tägiger intensivmedizinischer Überwachung und CT-A-Verlaufskontrolle beobachtet wurde.

**Figure 5:**
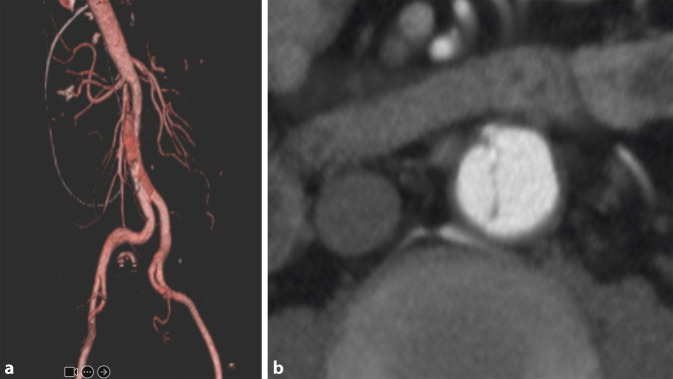

## Fazit für die Praxis

- Begleitende Gefäßverletzungen im Rahmen eines stumpfen Bauchtraumas treten deutlich seltener als bei penetrierenden abdominellen Verletzungen. Sie stellen komplexe Notfallsituationen dar, die ein multimodales Management erfordern und mit einer erhöhten Morbidität und Mortalität assoziiert sind.
- Entscheidend ist es, bei der primären Evaluation und klinischen Untersuchung des traumatisierten Patienten anhand des Verletzungsmechanismus und der betroffenen abdominellen Region (Zonen 1–3) an die begleitende Gefäßverletzung zu denken, danach zu suchen und die Diagnose, wenn möglich, bildgebend zu sichern.
- Die Diagnostik beinhaltet Sonographie und insbesondere die kontrastmittelunterstützte Dünnschicht-Computertomographie-Angiographie mit arterieller und später venöser Phase. Die Therapie ist aufgrund eines multimodalen Angebotes (konventionelle Chirurgie, endovaskuläre Therapie, Gerinnungsmanagement etc.) anspruchsvoll geworden.
- Der „moderne“ Gefäßchirurg, der endovaskulär und konventionell gleichermaßen ausgebildet ist, ist deshalb ein wichtiger klinischer Partner und sollte in der heutigen Zeit, in der eine fachübergreifende chirurgische Expertise aus einer Hand meist nicht mehr verfügbar ist, frühzeitig in das Behandlungskonzept eingebunden werden.
